# Supplementary material for: Effect of Antioxidant Supplementation on Milk Yield and Quality in Italian Mediterranean Lactating Buffaloes
Source: Animals (Basel). 2022 Jul 26;12(15):1903. doi: 10.3390/ani12151903 (PMC9330241; doi:10.3390/ani12151903)
Supplement: Supplementary file 1 [file animals-12-01903-s001.zip › animals-1833444-supplementary.pdf]

animals

Supplementary Materials

Effect of Antioxidant Supplementation on Milk Yield and Quality in Mediterranean Italian Lactating Buffaloes

Chiara Evangelista, Umberto Bernabucci, and Loredana Basiricò

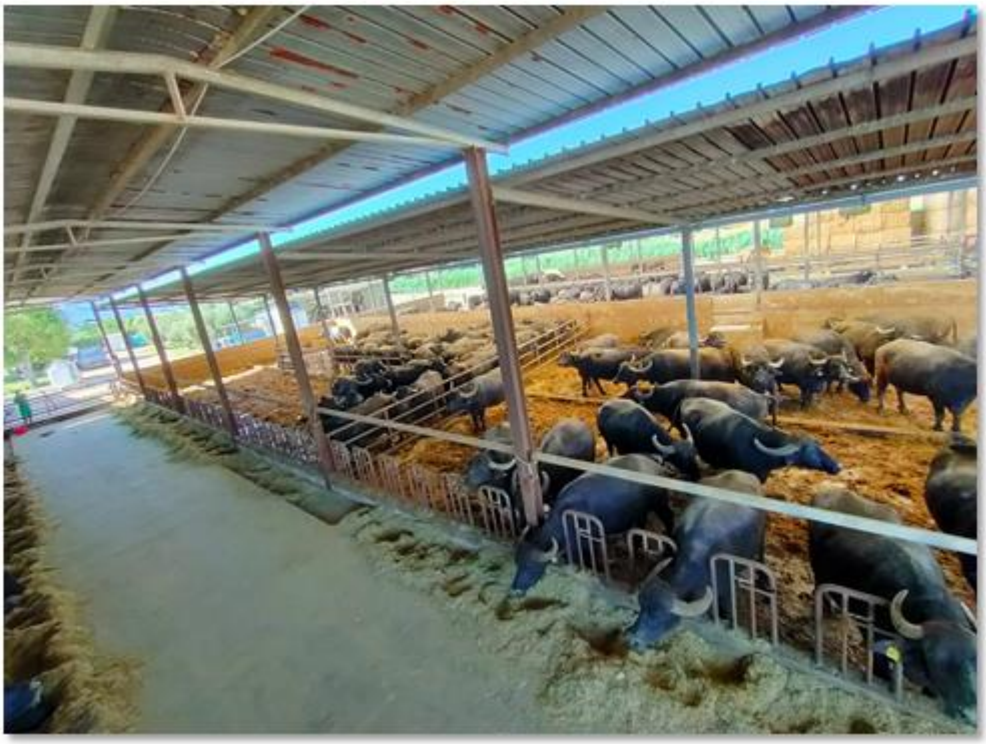

Figure S1. Effect of Antioxidant Supplementation on Milk Yield and Quality in Italian Mediterranean Lactating Buffaloes.
